# Comparative toxicity of menthol- and tobacco-flavored electronic cigarette constituents inducing inflammation, epithelial barrier dysfunction, and nicotinic acetylcholine receptor modulation in the absence of nicotine

**DOI:** 10.1016/j.toxrep.2026.102224

**Published:** 2026-02-14

**Authors:** Vidhi Pandya, Arni Bhatnagar, Kirby J. Beck, Thivanka Muthumalage

**Affiliations:** School of Health Sciences, Purdue University, West Lafayette, IN, USA

**Keywords:** Inflammation, Electronic cigarettes, Flavoring, Electronic nicotine delivery systems, Tobacco, Menthol, Acute lung injury, Chronic obstructive pulmonary disease

## Abstract

**Background:**

Menthol and tobacco-flavored nicotine delivery systems (ENDS) are widely used as safer alternatives to combustible cigarettes. These flavored products include constituents such as propylene glycol/vegetable glycerin (PG/VG), benzoic acid, acetoin, l-menthone, 98 % menthone, 2-isopropyl-N,2,3-trimethylbutanamide (WS-23), vanillin, and carvone. However, little is known about the potential adverse effects of the constituents in these flavored products.

**Rationale and hypothesis:**

We hypothesized that exposure to common constituents of tobacco- and menthol-flavored ENDS could elicit a lung-injurious response mediated by modulation of nicotinic acetylcholine receptors (α-nAChRs or CHRNA).

**Methods:**

Human bronchial epithelial cells, BEAS-2B, were treated with commonly used menthol and tobacco constituents on transwell inserts. Transepithelial barrier resistance (TEER) and millivolts (mV) across epithelial cells were measured over 24 h. To assess the elicited inflammatory response, cytokines IL-8 and IL-6 were quantified in the conditioned media. Cytotoxicity caused by these constituents was evaluated by acridine orange/propidium iodide (AO/PI) staining of the cells after 24 h. Alpha nicotinic receptor protein abundance (α1, α4, α5, and α7) was quantified by immunoblotting.

**Results:**

Epithelial integrity decreased over time, with significant decreases in TEER and voltage by ENDS constituents. A significant increase in IL-6 in conditioned media was observed in PG/VG, carvone, and WS-23 treated cells. Carvone-treated cells also elicited significantly elevated IL-8 in conditioned media. Further, increased α1, α4, α5, and α7 nAChR were seen in cells treated with PG/VG, acetoin, carvone, and WS-23.

**Conclusion:**

These findings suggested that common constituents in menthol- and tobacco-flavored ENDS induce lung inflammation, epithelial barrier dysfunction, and lung injury. Further, our data implicate potential lung disease pathogenesis via α-nAChRs modulation-mediated inflammation by exposure to these ENDS constituents, even in the absence of nicotine.

## Highlights

• Menthol- and tobacco-flavored e-liquid chemicals disrupt epithelial barrier integrity.
• Flavor constituents alone induce IL-6 and IL-8 inflammatory cytokine responses.
• Non-nicotine flavorants increase CHRNA5 and CHRNA7 abundance, typically nicotine-driven.
• This suggests oxidative or inflammatory upregulation independent of receptor agonism.
• Nicotine-free flavor chemicals mimic nicotine-like receptor and inflammatory effects.

## Graphical Abstract

**Figure:**
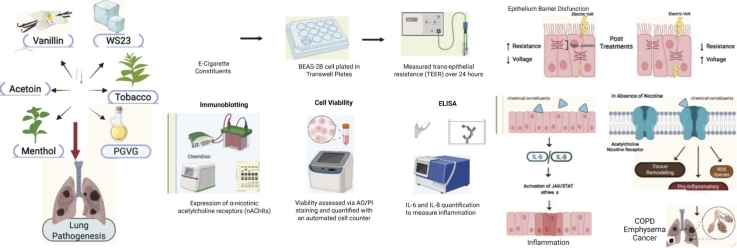

## 1 Background

The usage of electronic nicotine delivery systems (ENDS) or electronic cigarettes has increased in the past few decades. ENDS are often used as a smoking cessation product or a safer alternative to cigarettes. However, the use of ENDS products has become more prevalent among nonsmoking young adults [1]. Electronic Nicotine Delivery Systems (ENDS) consumption among middle and high school students was as high as 7.7 % or approximately 2.13 million students in 2023 [2]. Among the reported numbers, 89.4 % used flavored products, and 25.2 % participated in daily consumption of e-cigarettes [2]. The popularity of menthol and tobacco flavors is more prevalent among adults, around 22.3–31.8 % [3]. An overall increased use of e-cigarettes has been seen among youth participating in the PATH study from Wave 4 to Wave 5 [4].

Additionally, the 2020 FDA flavor ban on closed pod systems, with exemptions for menthol and tobacco, likely contributed to increased use of these flavoring agents [5] **(**Table 1**)**. This flavor ban was made exclusively for devices with pod and cartridge-based products, not for any disposable ENDS products. The use of menthol is more popular as it provides a soothing sensation upon inhalation and has antitussive properties [6]. A study indicated that adult e-cigarette users reported decreased irritation/harshness in the presence of menthol flavoring compared to unflavored products [7]. Along with those, higher concentrations of menthol are associated with analgesic effects that occur by TRPM8 sensory receptors (cold-receptors) activation leading to an increased subjective threshold of cough despite the ongoing irritation, increasing consumption of e-cigarettes and nicotine [6], [8].

**Table 1:** Constituents of menthol- and tobacco-flavored ENDS evaluated in this study.

**Chemical Name**	**Flavoring Element**	**Molecular Structure**	**Source**
Acetoin CAS 513-86-0	Creamy, Butter, Sweet		Vas et al. [9] McAdam et al. [10].
Benzoic Acid CAS 65-85-0	Unflavored		Cardenas et al. [11]. Tran et al. [12]
Carvone CAS 6485-40-1	Spearmint		Jabba et al. [13]. Tierney et al. [14].
L-Menthone CAS 14073-97-3	Mint		Tierney et al. [14]. Berkelhamer et al. [15]
Propylene Glycol(PG) CAS 57-55-6	Unflavored		Woodall et al. [16]. Kubica [17].
Vegetable Glycerin (VG) CAS 56-81-5			
Vanillin CAS 121-33-5	Vanilla, Sweet		Tierney et al. [14]. Berkelhamer et al. [15]. Krüsemann et al. [18].
N-2,3-Trimethyl-2-isopropylbutanamide (WS-23) CAS 51115-67-4	Koolada cooling agent		Leventhal et al. [19]. Wong et al. [20]. Omaiye et al. [21].
98 % Menthone CAS 10458-14-7	Mint, Cooling		Tierney et al. [14]. Berkelhamer et al. [15].

Tobacco-induced diseases such as chronic obstructive pulmonary disease (COPD), asthma, and idiopathic pulmonary fibrosis (IPD) are mediated via nicotinic receptors (nAchR proteins encoded by CHRNAs gene family) [22]. Nicotine in tobacco interacts with *α*7 nAchR, mediating lung injury [23]. Similarly, nicotine from electronic cigarettes is also known to bind to cholinergic nicotinic receptors, mediating addictive properties by activating the mesolimbic reward system [24]. However, it is unknown if other constituents in ENDS can modulate nicotinic receptors. To investigate the knowledge gap, we hypothesized that non-nicotine constituents in ENDS can modulate nAChR that mediates lung pathogenesis. Thus, we identified the key flavoring chemicals in Table 1 and assessed their ability to potentially induce lung injury via nAChRs using in-vitro assays. These receptors play a significant role in pulmonary epithelial cells, especially when exposed to foreign chemicals [22]. Nicotine from e-liquids is known to interact with many nAChRs and has been implicated in regulating the immune system, leading to inflammation [22]. These receptors also possess metabotropic functions in immune cells. nAChRs α7, α5, α4, and α1 were analyzed due to their prominent roles in the bronchial epithelial airway [25]. nAChR α7-dependent pathways also played a role in the prognosis for asthma and COPD [25]. Additionally, activation of α5 is linked to cell proliferation via TRPC3 channels [25] ome of these nAChRs have also displayed metabotropic signaling properties when bound to GTP-binding proteins that regulate cytokine expression [26].

Interleukin-6 (IL-6) is a known regulator of lung inflammation in addition to other inflammatory cytokines such as TNF*α*, IL1ß, and GM-CSF [27]. These cytokines recruit other leukocytes, inducing oxidative stress and lung inflammation [28], [29]. Increased cytokine levels have been observed in asthmatic patients' bronchoalveolar lavage fluid (BALF) compared to healthy non-smoking patients and stable non-asthmatic and asthmatic patients on mechanical ventilation [30]. The induction of IL-8, often synthesized by TNF*α*, has also been linked to conditions such as COPD, acute respiratory distress syndrome (ARDS) [29], and asthma [31], [32], [33]. Free IL-8 in bronchial tissue is also linked with patients experiencing severe asthma [34]. The pro-inflammatory effects of IL-8 have previously been linked to a stress or injury response [35]. Increased levels of IL-8 in BALF of many acute lung injury (ALI) patients have also been linked with an increase in mortality [36]. Like IL-6, increased levels of IL-8 in BALF have also been observed in several asthmatic patients [37], [9]. The risk of developing chronic obstructive pulmonary disease (COPD) is related to chronic inflammation in the lungs [38] through cytokine release and oxidative stress, which can lead to damaged lung parenchyma [39]. Additionally, chronic inflammation is associated with the expression of the NF-κB transcription factor that plays a vital role in inflammation-induced carcinogenesis [39]. It is also linked to the expression of pro-inflammatory cytokines such as IL-6 and IL-8 [32].

Exposure to vaping constituents can elicit an immune response and cause lung epithelial injury. Intercellular junctional complexes, including tight junctions (claudins and occludin), adherens junctions, and desmosomes, regulate epithelial permeability and maintain cellular polarity between adjacent cells. Barrier dysfunction in the epithelium has been linked with COPD, asthma, and pulmonary fibrosis [40]. The presence of tight junctions on the apical side of the transwells regulates paracellular transport between adjacent cells and adherens junctions, which aids cell-to-cell adhesion [40]. The use of the air-liquid interface (ALI) in this in-vitro study was best suited to simulate the respiratory epithelium and to assess barrier function, as well as cytokine release [40].

To test our hypothesis, in this study, we assessed the pulmonary toxicity of common flavoring chemicals and constituents in tobacco- and menthol-flavored ENDS–menthone, cooling agent (WS-23), carvone, acetoin, vanillin, benzoic acid, and PG/VG–utilizing a bronchial epithelial transwell-based model. The study will provide insights into the comparative toxicity of flavoring constituents in ENDS and their engagement in modulating nicotinic receptors.

## 2 Methods

### 2.1 Scientific rigor and reproducibility

We applied a robust, unbiased experimental design and data analysis approach throughout the study. We validated the methods and ensured reproducibility with repeated experiments. All methods are presented in detail with transparency. In vitro treatments were done in triplicates twice, using two different passages. TEER, ELISA, and immunoblotting assays were performed in a blinded manner and in 2–3 technical replicates. For all assays, laboratory-grade biological and chemical resources were purchased from commercial sources, and all primary and processed data are available upon request. Full western blot images are available in Supplementary figures (Supplementary Figs. S1–S5). Our methodologies, data, and results adhered to strict NIH reproducibility standards and scientific rigor.

### 2.2 Cell culture and treatments with flavoring chemicals

Human bronchial epithelial cells (BEAS-2B) (ATCC) were seeded on the apical side of 12 mm diameter polyester membrane transwell inserts with 0.4 µM pore size and 1.12 cm^2^ surface area (Corning #3460) in Dulbecco's Modified Eagle Medium/Nutrient Mixture F-12 1:1 (Gibco, Cat# 11320033) supplemented with 5 % fetal bovine serum (FBS), 15 mM HEPES, 1 % L-glutamine, and 1 % antibiotic-antimycotic. Cells were incubated at 37 °C and 5 % CO2. Once cells reached 80–85 % confluency, they were serum-deprived with 1 % FBS, and treatments were performed at 90–95 % confluency. The final volume in the apical layer containing cells was 750 µL and the final volume in the basolateral layer which did not contain cells was 2 mL before and after serum deprivation. Both apical (750 µL) and basolateral (2 mL) compartments were dosed to 100 µM by adding 2.75 µL of a 100 mM stock per insert (final ethanol <0.1 %). Transwells were employed with apical media as we wanted maintain a fixed dose and also obtain TEER measurements.

To perform flavoring chemical treatments analytical grade 100 mM stocks were prepared for L-menthone (Thermo Scientific, Catalog: A13679.18), 98 % menthone (Thermo Scientific, Catalog: 125411000), a 98 % racemic mixture of l and d-menthone and 2 % isomenthone. (R)-(-)-carvone (Thermo Scientific, Catalog: A13900.18), and acetoin (Sigma Aldrich, Catalog: 40127-U) by diluting respected volumes of them in 70 % ethanol. For the solids, such as vanillin (Thermo Scientific, Catalog: A11169.22), benzoic acid (Fisher Scientific, Catalog: A65–500), and WS-23 (N-2,3-trimethyl-2-isopropylbutanamide) (Sigma-Aldrich, Catalog: CDS003481), the respective masses were diluted in 70 % ethanol to make 100 mM flavor chemical stocks. The working concentration of 100 µM of the flavoring chemical of interest was achieved through diluting the stocks in 1 % FBS DMEM-F12 1:1 media. Equal parts of propylene glycol (Fisher Scientific, Catalog: P355-4) and glycerol (Fisher Scientific, Cat G31-1) were used to make a 0.25 % solution diluted in 1 % FBS DMDM-F12 media. Media pH was monitored qualitatively using phenol red throughout exposure; no gross color changes relative to controls were observed. Precise pH measurements were not performed. Osmolality was not independently measured; however, all treatments were prepared in identical complete media containing 15 mM HEPES buffer and applied at micromolar concentrations. Equivalent amount of ethanol 70 % ethanol was used as the vehicle (diluent) control to validate nontoxic effects at < 0.1 %. Hydrogen peroxide (100 µM) (Fisher Scientific, Catalog: H325–500) and TNFα (10 ng/µL) (Gibco, PHC3015) were used as positive controls for oxidative stress-driven injury and inflammation, respectively.

### 2.3 Lung epithelial barrier integrity and injury

To assess epithelial barrier integrity and injury the transepithelial electrical resistance (TEER) and the cell voltage (mV) was measured using EVOM2 Epithelial Volt-Ohmmeter (World Precision Instruments). At ∼85 % confluency, once the cells formed a monolayer with constant resistance overtime, cells were serum-deprived overnight and around 95 % confluency they were treated with chemicals of interest: l-menthone, 98 % menthone, carvone, WS-23, vanillin, benzoic acid, PG/VG, and acetoin as described above. TEER measurements were obtained using EVOM STX2 electrode chopstick electrodes at three predefined, evenly spaced positions around the membrane perimeter for each insert. The same positions were used consistently across timepoints and treatments. The average of the three readings was used for statistical analysis. Voltage (part of the TEER calculation, V = IR) and resistance readings were obtained before any chemical treatments for all plates, followed by readings at 6, 8, 20, and 24 h after the treatments, and were compared with untreated controls. TEER was calculated by multiplying the well surface area by its net resistance. A blank transwell insert with just DMEM:F12 media was used for background.

### 2.4 Cytotoxicity assessment

Twenty-four hours post-treatment, apical and basal media were collected from each well for cytokine assessment. Cells were then trypsinized (0.25 % Trypsin-EDTA, Gibco), and cell viability was assessed by AO/PI (Acridine Orange/Propidium Iodide) staining, and live, dead, and total cell counts were obtained using a DeNovix CellDrop automated cell counter (DeNovix, DE, USA).

### 2.5 Cytokine quantification by ELISA assay

The collected conditioned media from apical layer media were analyzed for the presence of Interleukin-6 (IL-6) (Invitrogen, Cat# KHC0061) and Interleukin-8 (IL-8) (Invitrogen, Cat# KHC0081) by ELISA using the Accuris Smart reader according to the manufacturer's protocol.

### 2.6 Immunoblotting

Harvested cells from each treatment group were lysed in RIPA buffer with complete™ protease inhibitor cocktail (Roche), and total protein was estimated using a BCA protein assay (Thermo Fisher, Cat# 23225). An equal amount of protein (5 µg) was added to 10 % SDS-PAGE gels, along with a Precision Plus Protein standard (Bio-Rad, Cat#1610376). Gel was transferred to PVDF membranes using the Trans-Blot Turbo (Bio-Rad) system, and transfer was confirmed by Ponceau S stain (Thermo Fisher Scientific, A40000279). Blots were blocked with 5 % non-fat dry milk in TBST (ASI, Cat# MB9696) for one hour at room temperature or overnight at 4 °C. The membranes were probed with specific primary antibodies: CHRNA1 (Abcam, Cat #AB308306), CHRNA4 (Abcam, Cat #AB124832), CHRNA5 (Abcam, Cat #AB259859), and CHRNA7 (Abcam, Cat #AB216485) overnight at 4 °C. The membranes were washed for 30 min with TBST and incubated with HRP-conjugated goat anti-rabbit secondary antibody (Abcam, Cat #AB6721) for an hour at room temperature. After the membranes were washed 3 times for 10 min each, they were subjected to chemiluminescence detection with SuperSignal West Femto (Thermo Fisher, Cat# 34094) using the Bio-Rad ChemiDoc XRS+ system. Subsequently, the blots were gently stripped for 10 min, washed, and reprobed with the remaining primary antibodies, followed by a loading control, ß-actin (Abcam, Cat #AB8227). Densitometry analysis was done using Image Lab software (Biorad) to analyze band intensity. Data were normalized to loading control β-actin.

### 2.7 Statistical analyses

All statistical analysis tests were managed on GraphPad Prism (Version 10.3.1). The TEER data were analyzed using a 2-way ANOVA with Šídák’s multiple comparisons test between the untreated control and TEER/mV measurements at 0, 6, 8, 20, and 24 h time points. Cytokine release and cytotoxicity were analyzed using one-way ANOVA with Dunnett’s multiple comparisons test comparing the untreated control to the various treatments. P < 0.05 was deemed statistically significant.

## 3 Results

### 3.1 Tobacco and menthol ENDS flavoring chemicals cause epithelial barrier dysfunction

**Fig. 1:**
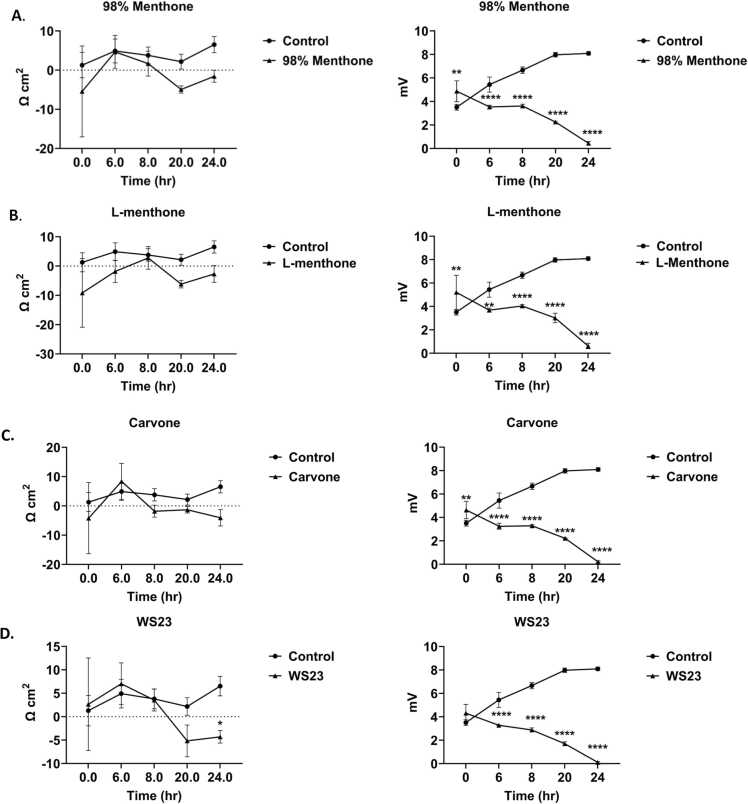

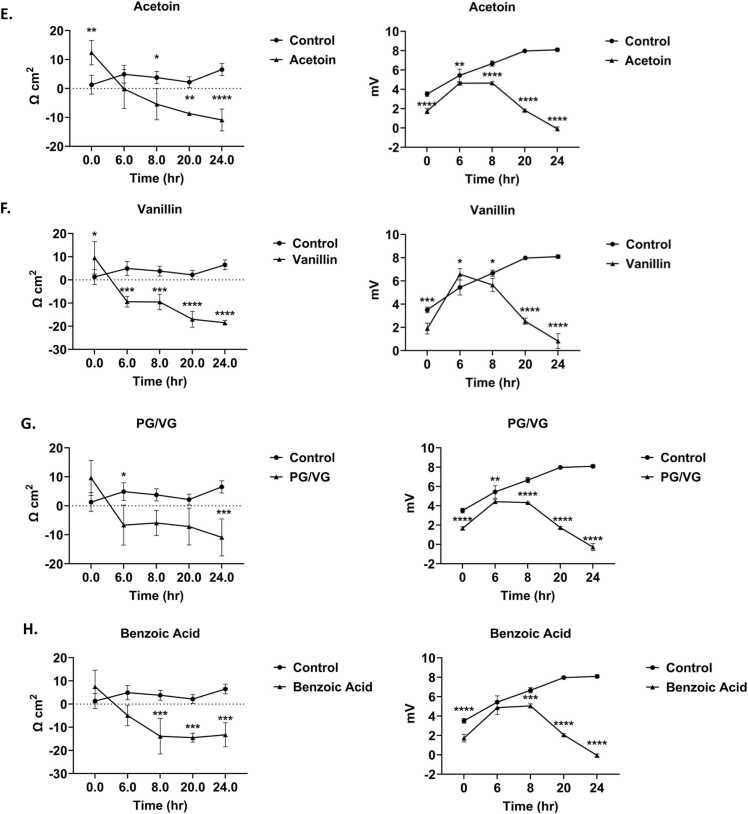
Menthol and tobacco flavoring chemicals caused BEAS-2B epithelial cell barrier dysfunction. BEAS-2B cells were grown in transwell inserts in complete medium. Once reached a monolayer and 80–85 % confluency, cells were serum deprived overnight. Around 90–95 % confluency, cells were treated with 100 μM (A) 98 % Menthone. (B) L-Menthone, (C). Carvone (D) WS-23 (E) Acetoin, (F) Vanillin, (G) PG/VG, and (H) Benzoic Acid. Transepithelial electrical resistance (TEER) and voltage (mV) data were collected pretreatment (0 hr), 6, 8, 20, and 24 hrs. following the treatments and the correlation of TEER and mV vs. time ± SEM are represented. *p < 0.05, **p < 0.01, ***p < 0.001, ****p < 0.0001 vs. untreated control., two-way ANOVA. N = 3 wells per chemical treatment.

All tested constituents–L-Menthone, 98 % Menthone, Carvone, WS-23, Vanillin, Benzoic Acid, and PG/VG, caused a decrease in epithelial barrier integrity compared to pretreatment and untreated control **(**Fig. 1**)**. The resistance of the cells exposed to L-menthone and 98 % menthone showed a decrease over time, but not statistically significant **(**Fig. 1 **A, B).** In comparison, L-menthone and 98 % menthone caused a more statistically significant reduction in voltage, with 98 % menthone showing more significance at the 6-hour time point when compared to L-menthone **(**Fig. 1 **A, B)**. Carvone caused a significant decrease in voltage over time **(**Fig. 1**C)**. Similarly, the cooling agent, WS-23, also had a significant decline in mV over time. WS-23 caused a similar decrease in TEER but the voltage drop at 24-h time point was statistically significant **(**Fig. 1**D).** Acetoin and Vanillin caused a significant decrease in both resistance and voltage over the course of 24 h with a slight improvement in mV during middle time points **(**Fig. 1**E, F)**. The primary vehicle component in e-liquids, PG/VG, caused a significant reduction in TEER and voltage at 24 h (Fig. 1**G**). Benzoic acid, used in nicotine salt products, caused a significant decrease in both resistance and voltage at 8, 20, and 24-h time points **(**Fig. 1**H)**. Ethanol as the solvent control showed similar effects to untreated control (P = 0.51 vs. control). Overall, either both TEER and mV or one of those parameters which is indicative of epithelial tight-junction health was adversely affected and declined over the 24-h period compared to untreated controls.

### 3.2 ENDS constituents elicited an inflammatory response in bronchial epithelial cells

**Fig. 2:**
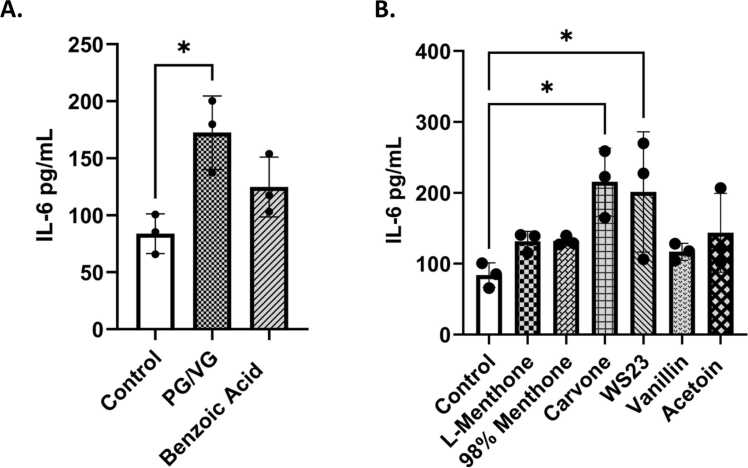
Menthol and tobacco flavoring constituents elicited an interleukin 6 cytokine response in lung epithelial cells. BEAS-2B cells cultured in transwells in complete media, 80–85 % confluency, and serum deprived overnight. Around 90–95 % confluency, cells were treated with 100µM L-Menthone, 98 % Menthone, Carvone, WS-23, Vanillin, Acetoin, Benzoic Acid, and PG/VG. Apical conditioned media was collected after the 24-h time point and IL-6 was quantified. (A) control, PG/VG, and Benzoic Acid-induced response, and (B) L-Menthone, 98 % Menthone, Carvone, WS-23, Vanillin, and Acetoin response compared to untreated control. IL-6 concentration in pg/mL ± SEM is represented, *p < 0.05. vs. control, one-way ANOVA. N = 3 wells per treatment.

**Fig. 3:**
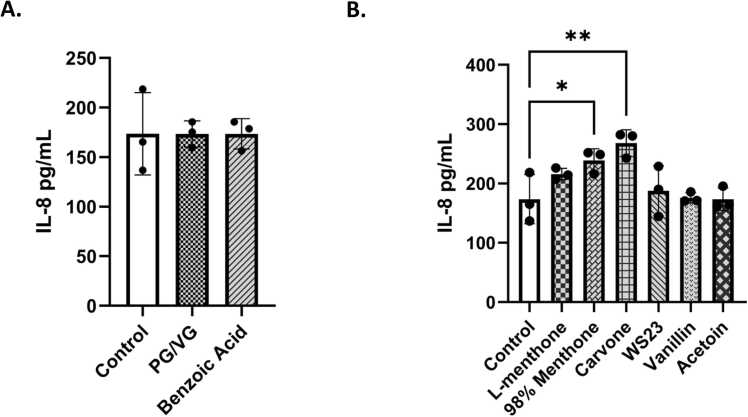
Menthol and tobacco flavoring constituents elicited an interleukin-8 cytokine response in lung epithelial cells. BEAS-2B cells cultured in transwells in complete media, 80–85 % confluency, and serum deprived overnight. Around 90–95 % confluency, cells were treated with 100 μM l-menthone, 98 % menthone, carvone, WS-23, vanillin, acetoin, benzoic acid, and PG/VG. Apical conditioned media was collected after the 24-h time point and IL6 was quantified. (A) control, PG/VG, and Benzoic Acid-induced response, and (B) l-menthone, 98 % menthone, carvone, WS-23, vanillin, and acetoin response compared to untreated control. IL-8 concentration in pg/mL ± SEM is represented, *p < 0.05, and **p < 0.01 vs. untreated control. one-way ANOVA. N = 3 wells per treatment.

Inflammatory mediators, IL-6 and IL-8, levels were measured in conditioned media to assess the elicited inflammatory response in lung epithelial cells by commonly found ENDS constituents in menthol and tobacco flavored products **(**Fig. 2**)**. PG/VG caused a significant IL-6 increase **(**Fig. 2**A).** IL-6 levels were increased in l-menthone, 98 % menthone, vanillin, and acetoin treated groups compared to the untreated controls **(**Fig. 2**B)**. Carvone and WS-23 elicited a significant response (*p < 0.05) (Fig. 2**B)**. Pro-inflammatory cytokine IL-8 was significantly increased by 98 % menthone (*p < 0.05) and carvone (**p < 0.05) compared to the untreated control cells **(**Fig. 3**)**.

### 3.3 Cytotoxicity assessment

**Fig. 4:**
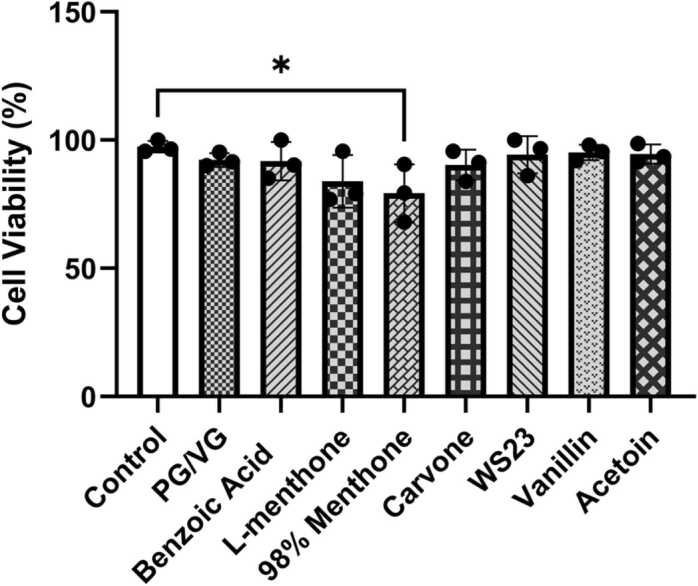
Menthol and tobacco flavoring constituents caused minimum cytotoxicity in BEAS-2B cells. BEAS-2B cells cultured in transwells in complete media, at 80–85 % confluency, and serum deprived overnight. Around 90–95 % confluency, Cells were treated with 100 μM l-menthone, 98 % menthone, carvone, WS-23, vanillin, acetoin, benzoic acid, and PG/VG. At the 24-h time point, cells were collected and stained with acridine orange and propidium iodide and the live, cell, and total cells were counted using CellDrop automatic cell counter. Cytotoxicity ± SEM is represented. *p < 0.05 vs. control, one-way ANOVA, N = 3 wells per treatment.

Compared to the untreated control, the cells treated with most constituents except 98 % menthone showed negligible cytotoxicity (<10 % cell death) **(**Fig. 4**)**. Ethanol (solvent control) and TNFα (inflammatory positive control) showed no toxicity compared untreated control (p > 0.05). The highest toxicity was observed in cells treated with 98 % menthone (∼19 %) (*p < 0.05). This suggests that for the tested chemicals, PG/VG, benzoic acid, carvone, WS23, vanillin, and acetoin, the tested concentration (100 μM) was sufficient to induce the observed responses without causing cell death.

### 3.4 Non-nicotine flavoring chemicals increased nicotinic acetylcholine receptors in bronchial epithelial cells

**Fig. 5:**
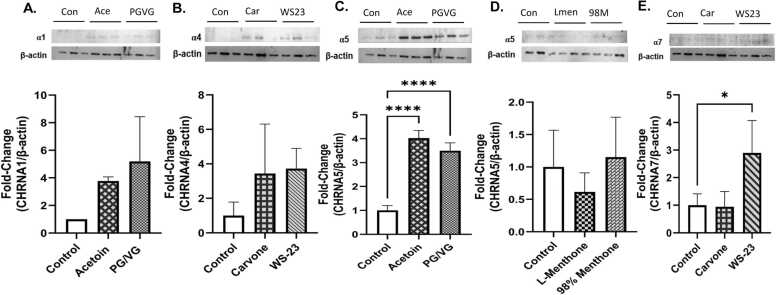
Menthol and tobacco flavoring constituents caused nicotinic acetylcholine receptor (nAchR) modulation in BEAS-2B lung epithelial cells. BEAS-2B cells cultured in transwells in complete media, 80–85 % confluency, and serum deprived overnight. Around 90–95 % confluency, cells were treated with 100 μM l-menthone, 98 % menthone, carvone, WS-23, vanillin, acetoin, benzoic acid, and PG/VG. At the 24-h time point, cells were collected, lysed, and after BCA protein estimation, 5 μg of protein were loaded to 10-well gel for SDS-gel electrophoresis. After cellulose membrane transfer and blocking, the membranes were probed with primary antibodies for nAchR1,4,5, and 7, with ß-actin loading control for normalization. The same membrane was sometimes re-probed up to 3 times with a different CHRNA. The blots with (A) Nicotinic Acetylcholine Receptors α1 expression with acetoin and PG/VG. (B) Nicotinic Acetylcholine Receptors α4 expression with carvone and WS-23. (C) Nicotinic Acetylcholine Receptors α5 expression with acetoin and PG/VG. (D) Nicotinic Acetylcholine Receptors α5 expression with l-menthone and 98 % menthone. (E) Nicotinic Acetylcholine Receptors α7 expression with carvone and WS-23. All respective CHRNA bands ß-actin are shown with their densitometry fold-change ± SEM. *p < 0.05 and ****p < 0.0001 vs. control, one-way ANOVA. N = 3 wells per chemical. Full blots are shown in the Supplementary section (Fig. S1–S5).

Prepared lysates of menthol and tobacco constituent treated cells were used to perform immunoblotting to analyze protein abundance of nicotinic acetylcholine receptors 1–7(nAchRs) compared to untreated controls. The data was normalized via housekeeping protein beta-actin **(**Fig. 5**)**. CHRNA1 was increased by acetoin and PG/VG treatments (not significant) **(**Fig. 5**A)**. CHRNA4 was increased by carvone and acetoin (not significant) **(**Fig. 5**B)**. A significant increase in the CHRNA5 for cells treated with acetoin and PG/VG compared to the control group (p****<0.0001) while menthone and 98 % menthol showed no change **(**Fig. 5**C, D)**. Additionally, a significant increase in protein abundance for CHRNA7 was observed in cells treated with WS-23 (*p < 0.05) but carvone did not cause any changes. These data suggest that CHRNA 5 and 7 were more affected compared to other measured CHRNAs by the tested chemicals.

## 4 Discussion

Electronic cigarettes are becoming widely popular, and menthol and tobacco flavors are used as a safer alternative for adult users and a cessation product to combustible cigarettes [8], [41]. While these flavors constitute numerous chemicals, key chemicals that are commonly used in menthol- and tobacco-flavored ENDS products such as acetoin, vanillin, 98 % menthone, WS-23, carvone, and base liquid constituent PG/VG and benzoic acid were selected to test the hypothesis that low concentrations in liquids can cause lung epithelial barrier dysfunction, elicit an inflammatory response, and modulate nicotinic acetylcholine receptors independent of nicotine. Similar to our study, these chemicals have been used due to their prevalence in several studies involving the characterization of e-liquids, specifically, the ones that were menthol and tobacco flavored **(**Table 1**)**.

Our in-vitro data demonstrated acute adverse effects on epithelial barrier function by decreasing TEER and mV values. While, acetoin, vanillin, WS-23, and PG/VG caused a significant decrease in both TEER and mV across the membrane, suggesting the disruption of the tight junctions by the chemicals, l-menthone, 98 % menthone, and carvone caused a severe decline in voltage across the membrane suggesting, even though the tight junctions were not impaired or leaking, epithelial polarity, metabolism, and ion transport have been adversely affected as before structural breakdown [42], [43] These findings corroborate with our in-vitro studies where ENDS aerosols have been shown to cause barrier dysfunction [44], [45].

Our cytotoxicity data demonstrated the concentration we tested in the study at 100 μM was sufficient to induce a response but did not cause significant cell death, except for 98 % menthone. Consistent with these results, other studies have shown that menthol flavors have greater toxicity in their BEAS-2B, 16HBE, and CALU3 upon ENDS chemical and aerosol exposures [44], [45], [46], [47].

Our data showed increased IL-6 and IL-8 pro-inflammatory cytokine response by PG/VG, carvone, WS-23, l-menthone, and 98 % menthone. These cytokines can initiate and amplify the inflammatory response by recruiting other immune cells, such as neutrophils, in the acute phase, which are often increased in acute lung injury, asthma, and infections [30], [48]. Even though we saw a significant cell death by 98 % menthone, we observed significantly elevated cytokine levels, suggesting the cell death occurred around the 20 h time point based on declined TEER and mV drop around the same time.

Acetoin is a known precursor of diacetyl [9], a compound found in e-liquids whose inhalation has been associated with bronchiolitis obliterans in many flavoring manufacturing workers [49]. Storage of acetoin in oxidative conditions, such as the presence of PG/VG or nicotine, has shown diacetyl formation within normal commercial shelf life [9]. Benzoic acid, which is often used in nicotine salt formulations, has been reported to decrease overall TEER values following a 24-h exposure to 3 % PG/VG in human bronchiolar epithelial cells [16]. Consistent with our cytokine results, exposure to aerosolized PG/VG in normal human bronchial epithelial cells (HBEC) cells showed increased mRNA markers for both IL-6 and IL-8, further supporting our findings [50]. These in-vitro studies corroborate the adverse cellular effects we observed by benzoic acid and PG/VG, such as barrier dysfunction and inflammation.

In our study, WS-23, a synthetic cooling agent added to vaping products, showed similar comparative toxicity to other menthol analogs. We observed barrier dysfunction, IL-6 elevation, and CHRNA7 increase by WS-23. Our data and other in-vitro studies on airway epithelial cells with aerosolized WS-23 and nicotine showed an increased IL-6 following a 72-h exposure, suggesting that synthetic cooling agents induce lung inflammation [51]. Our study demonstrated that WS-23 containing “ice” vaping products may pose similar pulmonary toxicity in comparison to other menthol additives [19].

Nicotinic acetylcholine receptors (nAchRs) are cholinergic and are typically activated by binding to their ligands, nicotine and acetylcholine, and are known to be involved in lung disease pathogenesis [22], [25], [52], [53]. In BEAS-2B lysates, we determined the CHRNA subunit dysregulated expression after treatment with the chemicals. Our data showed that PG/VG, acetoin, and WS-23 significantly augmented the α5 and α7 subunits compared to untreated counterparts, suggesting receptor desensitization, immunomodulation, and increased risk for inflammatory diseases like COPD. Supporting our data, α7 receptor-mediated pathways have been shown to play a role in airway remodeling and obstruction, contributing to the progression of COPD [22]. Additionally, it also mediates pathways responsible for lung cancer in the presence of nicotine [54]. These subunits are involved in JAK/STAT, ERK1/2, and PI3K/Akt mechanisms of metabolism, proliferation, and inflammation [53]. We also demonstrated increased α1 and α4 involved by PG/VG, acetoin, carvone, and WS-23, which may suggest stress response and cytokine modulation. CHRNA1(α1), neuromuscular receptor increases may also be due to abnormal receptor remodeling. Overall, increased CHRNA expression demonstrated the increased sensitivity to these non-nicotinic chemicals, potentially mediating lung diseases similar to nicotine receptor signaling. This indicates the possible pathogenesis of pulmonary diseases such as emphysema/COPD with e-cigarette use, even in the absence of nicotine. Further investigation is required to learn more about the mechanism of nAchR receptor signaling in lung disease pathogenesis by non-nicotinic receptors.

Several ENDS constituents evaluated here (e.g., WS-23, glycerin, propylene glycol) have been examined in animal models and, for some constituents such as propylene glycol, in controlled human exposure studies. However, differences in exposure paradigms, delivered dose, metabolic capacity, and endpoint sensitivity limit direct quantitative comparisons across in vitro, in vivo, and human systems. Thus, this study was a controlled comparative epithelial hazard-identification and mechanistic screen of individual ENDS-flavor constituents (in the absence of nicotine), designed to complement existing in vivo and clinical evidence. Chemical analyses of commercial ENDS liquids report many flavorants/synthetic coolants at millimolar concentrations in reservoir fluids. To enable side-by-side comparison across multiple constituents while avoiding overt cytotoxicity, we selected 100 µM as a single screening concentration that reliably produced measurable changes in epithelial barrier and inflammatory endpoints in our model. While study was not quantitative dosimetry/IVIVE assessment. The concentrations tested were selected to be aerosol-relevant for comparative hazard screening based on reported reservoir concentrations and prior in vitro ENDS toxicology literature; however, we did not perform quantitative in vitro-to-in vivo extrapolation, and true human airway epithelial exposure will vary with device type, puffing behavior, transfer efficiency, deposition, airway surface liquid dilution, clearance, mucosal response, and biotransformation.

This study used BEAS-2B bronchial epithelial cells in transwell culture as a reproducible screening platform; however, BEAS-2B exhibit a basal-like, non-fully differentiated phenotype and relatively low baseline TEER, which limits interpretation of absolute barrier values and favors interpretation of relative within-model changes. In addition, immortalized epithelial monocultures do not recapitulate the cellular diversity of the human lung (e.g., ciliated, secretory, basal subtypes, immune cells, fibroblasts, endothelial cells) or metabolic interactions (e.g., epithelial–immune crosstalk, xenobiotic metabolism/biotransformation, mucociliary clearance). As treatments were performed to assess individual constituents induced responses these findings should be interpreted as hazard-identification/mechanistic signals.

In summary, our data showed that commonly used non-nicotine-constituents of menthol and tobacco-flavored ENDS modulate nicotinic receptor expression on lung epithelial cells, cause lung epithelial barrier dysfunction–by altering polarization, hindering ion transport, and by disrupting the tight-junctions–and elicit cytokine responses such as IL-6 and IL-8 which are involved in innate immunity and acute lung injury responses.

## 5 Conclusion

Our data support the hypothesis that chemicals in e-liquids cause epithelial barrier dysfunction, induce lung inflammation, and alter nicotinic receptor signaling pathways, leading to lung diseases. These findings were not a result of pH changes in the in-vitro system. The concentration of the constituents is a human relevant dose treatment to aerosolized chemical from e-liquids. The findings provide insights into regulating constituents and their concentrations to achieve potentially safer levels. While these results do not directly establish in vivo disease outcomes, they provide mechanistic and hazard-identification insight into how individual e-liquid constituents may contribute to airway epithelial dysfunction. These findings underscore the importance of evaluating and regulating specific ENDS constituents and their concentrations to inform the development of potentially safer formulations and guide future in vivo and human-relevant studies.

## List of Abbreviations

COPD - Chronic obstructive pulmonary disease

ELISA - Enzyme-linked immunosorbent assay

PG/VG - Propylene glycol/Vegetable glycerol

AO/PI - Acridine orange/Propidium iodide

IL-6 - Interleukin-6

IL-8 - Interleukin-8

ENDS - Electronic nicotine delivery systems

PATH - Population Assessment of Tobacco and Health

ARDS - Acute respiratory distress syndrome

TNF - Tumor necrosis factor

BALF - Bronchoalveolar lavage fluid

ALI - Acute lung injury

ALI - Air-liquid interphase

NF-κB - Nuclear factor kappa B

FBS - Fetal bovine serum

HEPES - 4-(2-hydroxyethyl)piperazine-1-ethanesulfonic acid

DMEM - Dulbecco's Modified Eagle Medium

TEER - Transepithelial electrical resistance

EDTA - Ethylenediaminetetraacetic acid

HBEC - Primary human bronchial epithelial cells

CHRNA/nAchR - Nicotinic Acetylcholine Receptors

## CRediT authorship contribution statement

**Pandya Vidhi:** Writing – review & editing, Writing – original draft, Visualization, Methodology, Investigation, Formal analysis, Data curation. **Beck Kirby:** Writing – review & editing, Writing – original draft, Visualization, Validation, Methodology, Investigation, Formal analysis, Data curation, Conceptualization. **Thivanka Muthumalage:** Writing – review & editing, Validation, Supervision, Software, Resources, Project administration, Methodology, Investigation, Funding acquisition, Conceptualization. **Arni Bhatnagar:** Writing – review & editing, Writing – original draft, Visualization, Validation, Methodology, Formal analysis, Data curation.

## Funding

This study was supported by the 10.13039/100000002National Institutes of Health (NIH), National Institutes of Environmental Health Sciences (NIEHS) award R00S033835.

## Declaration of conflicts of interest

The authors declare that the research was conducted in the absence of any commercial or financial relationships that could be construed as a potential conflict of interest, and no potential conflicts of interest with respect to the authorship, and/or publication of this article.

## Competing interests

None.

## Declaration of Competing Interest

The authors declare that they have no known competing financial interests or personal relationships that could have appeared to influence the work reported in this paper.

## Data Availability

Data will be made available on request.
