# Supplementary material for: Comparative toxicity of menthol- and tobacco-flavored electronic cigarette constituents inducing inflammation, epithelial barrier dysfunction, and nicotinic acetylcholine receptor modulation in the absence of nicotine
Source: Toxicol Rep. 2026 Feb 14;16:102224. doi: 10.1016/j.toxrep.2026.102224 (PMC12989985; doi:10.1016/j.toxrep.2026.102224)
Supplement: Supplementary file 1 — Supplementary material [file mmc1.docx]

**Title: Comparative toxicity of menthol- and tobacco-flavored electronic cigarette constituents inducing inflammation, epithelial barrier dysfunction, and nicotinic acetylcholine receptor modulation in the absence of nicotine**

**Pandya et al, 2026**

**Supplementary Figures S1-S9**

Gel 2- Alpha 1

**Figure S1**. Menthol and tobacco flavoring constituents caused nicotinic acetylcholine receptor (CHRNA) modulation in BEAS2B lung epithelial cells. BEAS-2B cells cultured in transwells were treated with 100 μM Acetoin and PG/VG. At the 24 hour time point, cells were collected, lysed, and after BCA protein estimation, 5-10 μg of protein were loaded to 10-well gel for SDS-gel electrophoresis. After cellulose membrane transfer and blocking, the membranes were probed with primary antibodies for CHRNA 1 with beta-actin loading control for normalization. The same membrane was later re-probed for CHRNA 4, 5, and 7. Western blot analysis of blot with Nicotinic Acetylcholine Receptors Alpha 1 reveals increased expression of Acetoin and PG/VG at 54.4 kDa. The respective CHRNA bands are quantified using beta actin via densitometry fold-change. No significance compared to the control group using one-way ANOVA. N=3 wells per chemical.

Gel 2- Alpha 5

**Figure S2.** Menthol and tobacco flavoring constituents caused nicotinic acetylcholine receptor (CHRNA) modulation in BEAS2B lung epithelial cells. BEAS2B cells cultured in transwells were treated with 100 μM Acetoin and PG/VG. At the 24 hour time point, cells were collected, lysed, and after BCA protein estimation, 5-10 μg of protein were loaded to 10-well gel for SDS-gel electrophoresis. After cellulose membrane transfer and blocking, the membranes were probed with primary antibodies for CHRNA 5 with beta-actin loading control for normalization. The same membrane was later re-probed for 1, 4, and 7 CHRNA. Western blot analysis of blot with Nicotinic Acetylcholine Receptors Alpha 5 reveals increased expression of Acetoin and PG/VG at 53.1 kDa. The respective CHRNA bands are quantified using beta actin via densitometry fold-change. ****p<0.0001 compared to the control group using one-way ANOVA. N=3 wells per chemical.

Gel 3- Alpha 5

**Figure S3.** Menthol and tobacco flavoring constituents caused nicotinic acetylcholine receptor (CHRNA) modulation in BEAS2B lung epithelial cells. BEAS-2B cells cultured in transwells were treated with 100 μM L-Menthone and 98% Menthone. At the 24 hour time point, cells were collected, lysed, and after BCA protein estimation, 5-10 μg of protein were loaded to 10-well gel for SDS-gel electrophoresis. After cellulose membrane transfer and blocking, the membranes were probed with primary antibody for CHRNA 5 with beta-actin loading control for normalization. The same membrane was later re-probed for 1, 4, and 7 CHRNA. Western blot analysis of blot with Nicotinic Acetylcholine Receptors Alpha 5 reveals decreased expression of L-Menthone and increased expression of 98% Menthone at 53.1 kDa. The respective CHRNA bands are quantified using beta actin via densitometry fold-change. No significance compared to the control group using one-way ANOVA. N=3 wells per chemical.

Gel 4- Alpha 4

**Figure S4**. Menthol and tobacco flavoring constituents caused nicotinic acetylcholine receptor (CHRNA) modulation in BEAS2B lung epithelial cells. BEAS-2B cells cultured in transwells were treated with 100 μM Carvone and WS-23. At the 24 hour time point, cells were collected, lysed, and after BCA protein estimation, 5-10 μg of protein were loaded to 10-well gel for SDS-gel electrophoresis. After cellulose membrane transfer and blocking, the membranes were probed with primary antibodies for CHRNA 4 with beta-actin loading control for normalization. The same membrane was later re-probed for 1, 5, and 7 CHRNA. Western blot analysis of blot with Nicotinic Acetylcholine Receptors Alpha 4 reveals increased expression of Carvone and WS-23 at 70.0 kDa. The respective CHRNA bands are quantified using beta actin via densitometry fold-change. No significance compared to the control group using one-way ANOVA. N=3 wells per chemical.

Gel 4- Alpha 7

**Figure S5.** Menthol and tobacco flavoring constituents caused nicotinic acetylcholine receptor (CHRNA) modulation in BEAS2B lung epithelial cells. BEAS-2B cells cultured in transwells were treated with 100 μM Carvone and WS-23. At the 24 hour time point, cells were collected, lysed, and after BCA protein estimation, 5-10 μg of protein were loaded to 10-well gel for SDS-gel electrophoresis. After cellulose membrane transfer and blocking, the membranes were probed with primary antibodies for CHRNA 7 with beta-actin loading control for normalization. The same membrane was later re-probed for 1, 4, and 5 CHRNA. Western blot analysis of blot with Nicotinic Acetylcholine Receptors Alpha 7 reveals increased expression of WS-23 at 56.4 kDa. The respective CHRNA bands are quantified using beta actin via densitometry fold-change. *p<0.05 compared to the control group using one-way ANOVA. N=3 wells per chemical.

**Figure S6.** Menthol and tobacco flavoring constituents caused minimal cytotoxicity in BEAS-2B cells. BEAS-2B cells cultured in transwells in complete media, at 80-85% confluency, were serum-deprived overnight. At 90-95% confluency, cells were treated with 100 µM hydrogen peroxide, TNF-α, ethanol, L-menthone, 98% menthone, carvone, WS-23, vanillin, acetoin, benzoic acid, and PG/VG. At the 24 hr time point, cells were collected and stained with acridine orange and propidium iodide and the live, cell, and total cells were counted using CellDrop automatic cell counter. Cytotoxicity±SEM is represented. *p<0.05 vs. control, one-way ANOVA, N=3 wells per treatment.

**Figure S7**. Interleukin-6 cytokine response in lung epithelial cells after exposure to chemical constituents. BEAS-2B cells cultured in transwells in complete media, at 80-85% confluency, were serum-deprived overnight. At 90-95% confluency, cells were treated with 100 μM hydrogen peroxide, TNF-α, ethanol, L-menthone, 98% menthone, carvone, WS-23, vanillin, acetoin, benzoic acid, and PG/VG. Apical conditioned media were collected after the 24-hour time point, and IL-6 was quantified. The positive controls, PG/VG, benzoic acid, L-menthone, 98% menthone, carvone, WS-23, vanillin, and acetoin response were compared to the untreated control. IL-6 concentration in pg/mL ± SEM is represented, *p<0.05. vs. control, one-way ANOVA. N=3 wells per treatment.

**Figure S8**. Interleukin-8 cytokine response in lung epithelial cells after exposure to chemical constituents. BEAS-2B cells cultured in transwells in complete media, at 80-85% confluency, were serum-deprived overnight. At 90-95% confluency, cells were treated with 100 μM hydrogen peroxide, TNF-α, ethanol, L-menthone, 98% menthone, carvone, WS-23, vanillin, acetoin, benzoic acid, and PG/VG. Apical conditioned media were collected after the 24-hour time point, and IL-8 was quantified. The positive controls, PG/VG, benzoic acid, L-menthone, 98% menthone, carvone, WS-23, vanillin, and acetoin response were compared to the untreated control. IL-8 concentration in pg/mL ± SEM is represented, *p<0.05. vs. control, one-way ANOVA. N=3 wells per treatment.

**Figure S9**. BEAS-2B epithelial cell barrier dysfunction by chemical constituent treatments. BEAS2B cells were grown in transwell inserts in a complete medium. Once 80-85% of confluency was achieved, the cells were serum-deprived overnight. At 90-95% confluency, cells were treated with 100 uM (A) Ethanol, (B) Hydrogen Peroxide, and (C). TNF-α. Transepithelial electrical resistance (TEER) data were collected during pretreatment (0 hr), 6, 8, 20, and 24 hrs. following the treatments, and the correlation of TEER vs. time ± SEM is represented. *p<0.05, **p<0.01, ***p<0.001, ****p<0.0001 vs untreated control., one-way ANOVA. N=3 wells per chemical treatment.
