# Supplementary material for: Comparative toxicity of menthol- and tobacco-flavored electronic cigarette constituents inducing inflammation, epithelial barrier dysfunction, and nicotinic acetylcholine receptor modulation in the absence of nicotine
Source: Toxicol Rep. 2026 Feb 14;16:102224. doi: 10.1016/j.toxrep.2026.102224 (PMC12989985; doi:10.1016/j.toxrep.2026.102224)
Supplement: Supplementary file 2 — Supplementary material [file mmc2.pptx]

## Slide 1
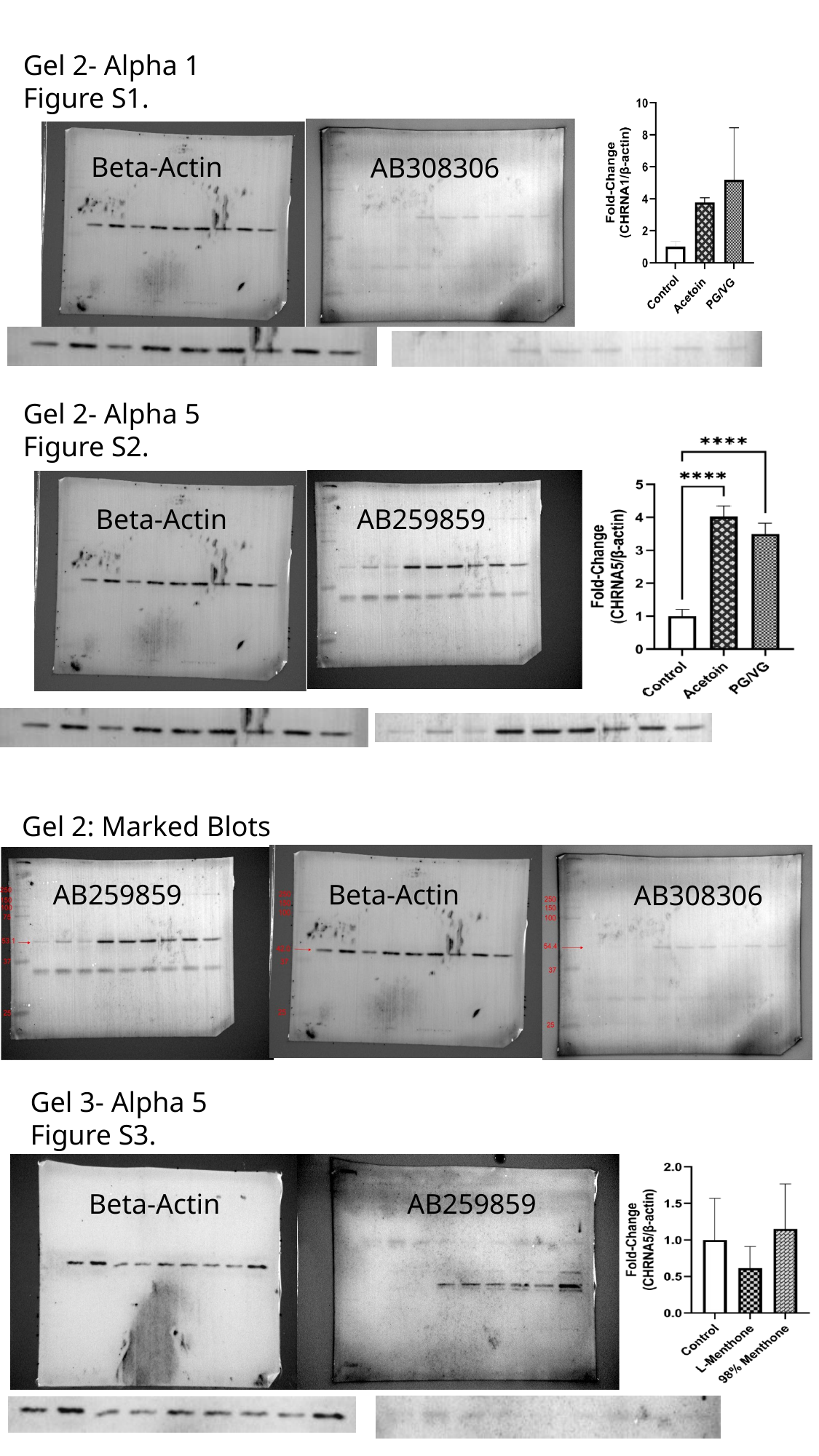

Gel 2- Alpha 1
Figure S1.
Beta-Actin
AB308306
Gel 2- Alpha 5
Figure S2.
Beta-Actin
AB259859
Gel 2: Marked Blots
AB259859
Beta-Actin
AB308306
Gel 3- Alpha 5
Figure S3.
AB259859
Beta-Actin

## Slide 2
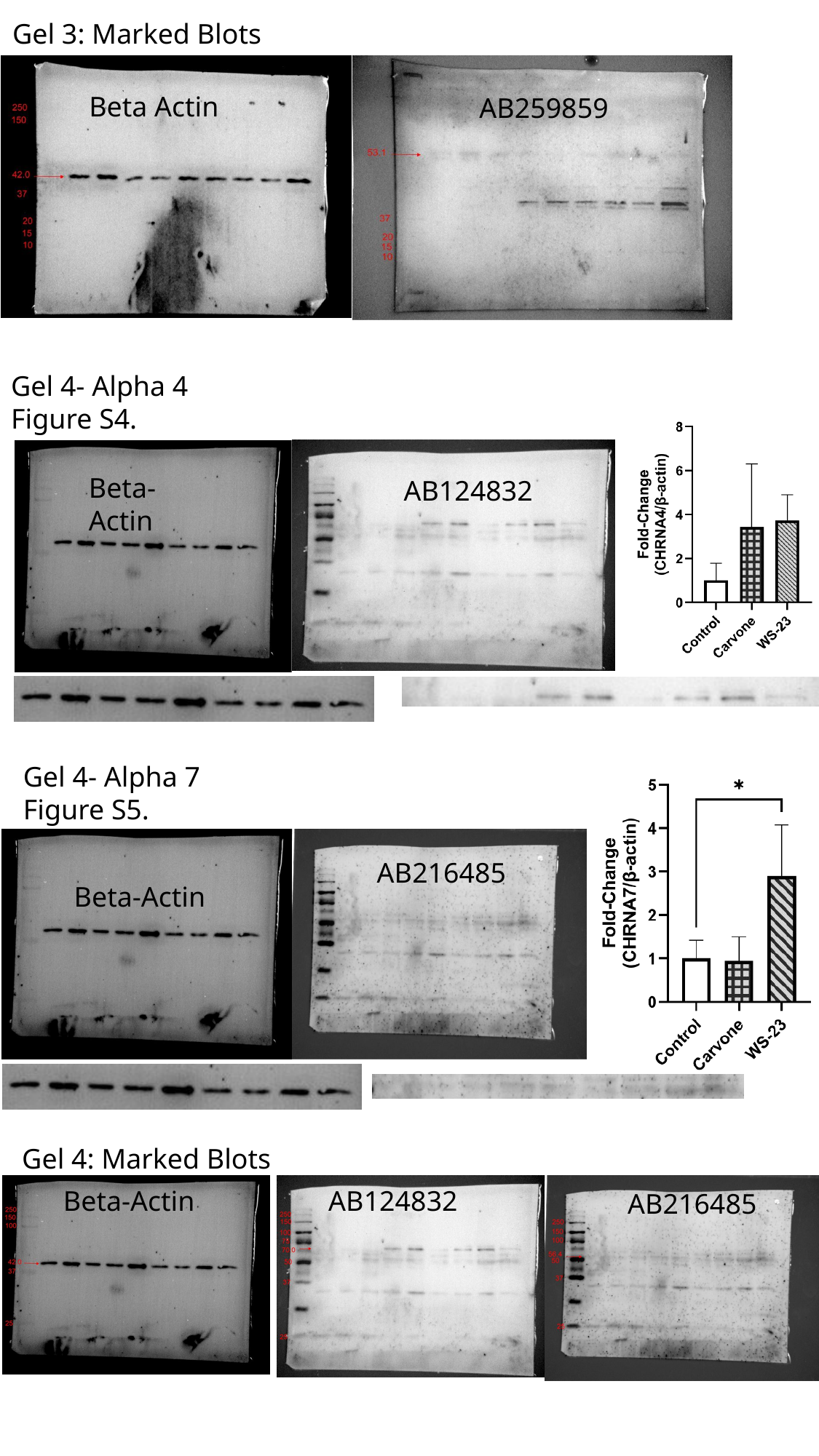

Gel 3: Marked Blots
Beta Actin
AB259859
Gel 4- Alpha 4
Figure S4.
Beta-Actin
AB124832
Gel 4- Alpha 7
Figure S5.
AB216485
Beta-Actin
Gel 4: Marked Blots
Beta-Actin
AB124832
AB216485

## Slide 3
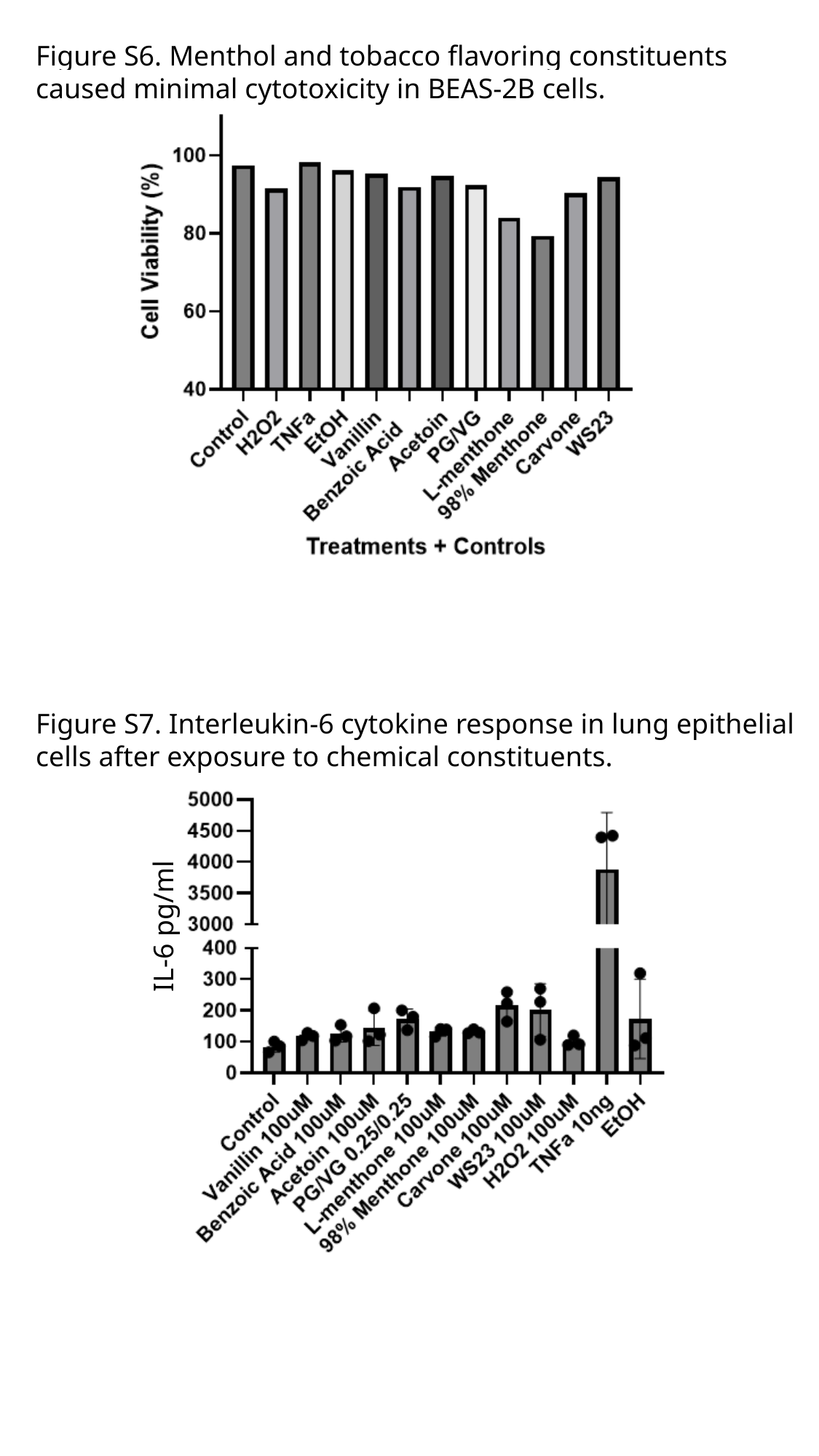

Figure S6. ​Menthol and tobacco flavoring constituents caused minimal cytotoxicity in BEAS-2B cells.
Figure S7. Interleukin-6 cytokine response in lung epithelial cells after exposure to chemical constituents.
IL-6 pg/ml

## Slide 4
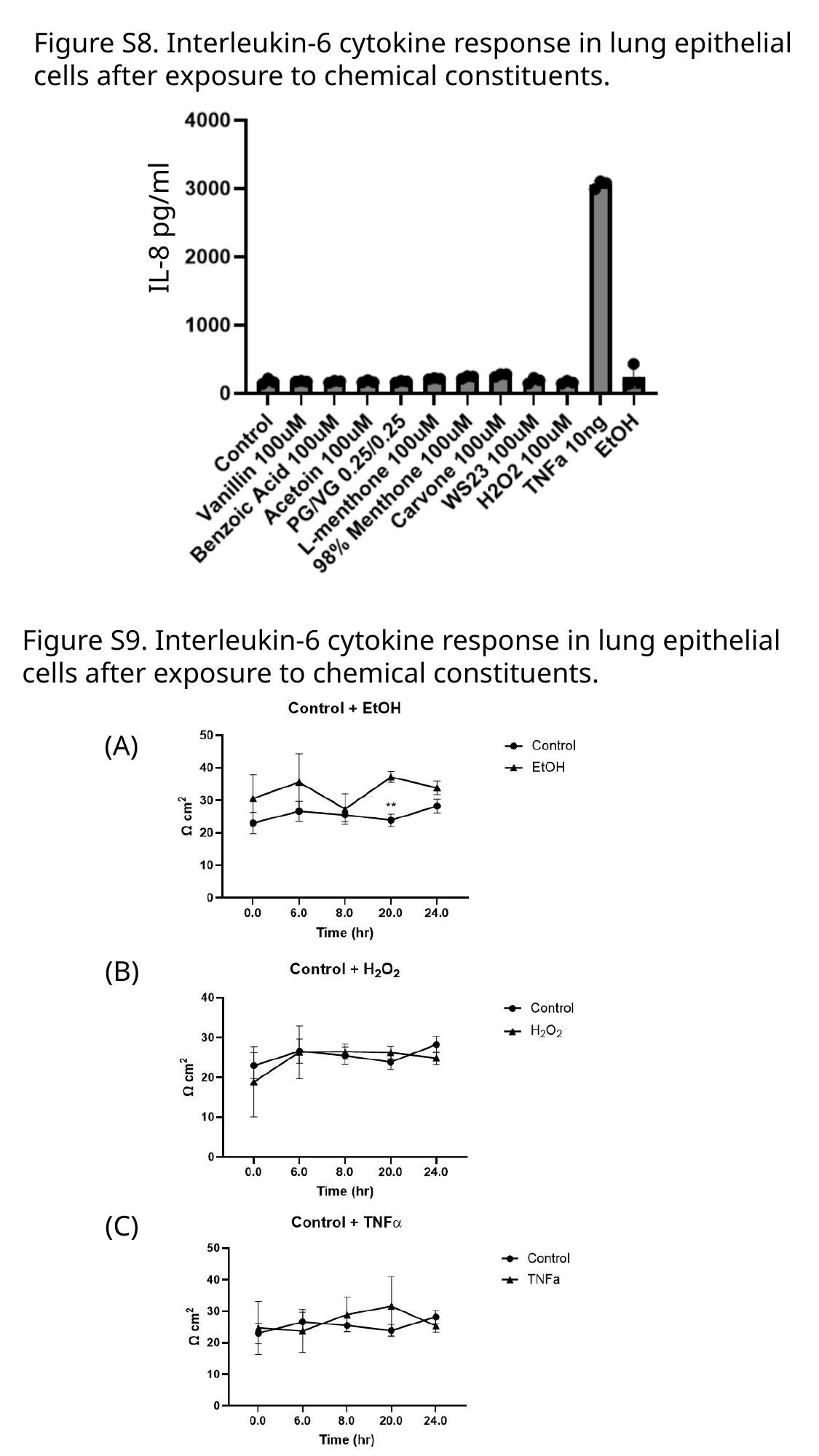

Figure S8. Interleukin-6 cytokine response in lung epithelial cells after exposure to chemical constituents.
IL-8 pg/ml​
Figure S9. Interleukin-6 cytokine response in lung epithelial cells after exposure to chemical constituents.
(A)
(B)​
(C)​
